# Multi-Source Common-View Disciplined Clock: A Fail-Safe Clock for Critical Infrastructure Systems

**DOI:** 10.6028/jres.126.041

**Published:** 2022-01-10

**Authors:** Michael A. Lombardi

**Affiliations:** 1 National Institute of Standards and Technology, Boulder, CO 80305, USA

**Keywords:** Coordinated Universal Time, critical infrastructure systems, synchronization, time transfer

## Abstract

The multi-source common-view disciplined clock (MSCVDC) is a recent NIST invention designed to support critical infrastructure timing systems that require a verifiably accurate and fail-safe clock. This paper introduces the MSCVDC, provides a technical description of how it works, and discusses its reliability, redundancy, security, and performance. It also discusses the possibility of a commercially available MSCVDC product.

## 1 Introduction

Accurate and verifiable time is a requirement for many critical infrastructure systems, including systems operated by banks, stock exchanges, telecommunication networks, and electric power utilities. These systems cannot function properly without accurate time signals and timing system failures can have serious consequences; with the potential implications including economic loss, reduced safety and security, and even loss of human life. Critical infrastructure systems usually obtain the accurate time they need from disciplined clocks, or clocks that are synchronized by external reference signals. In nearly all cases in the United States, the external reference for the disciplined clocks consists of signals broadcast by Global Positioning System (GPS) satellites. This has led to a reliance and dependency on GPS that has potentially huge consequences if GPS were to become unavailable [1, 2].

The multi-source common-view disciplined clock (MSCVDC), based on an invention patented by the National Institute of Standards and Technology (NIST) [3], was designed to support critical infrastructure systems that require a very accurate and very reliable clock that originates from a verifiable time source. To be more specific, “very accurate” means that an MSCVDC can easily keep time within ±1 µs of Coordinated Universal Time (UTC), and “very reliable” means that it has enough redundancy to continue to be accurate even when one or more parts of the clock have failed or deteriorated. The MSCVDC gets the accuracy it needs by synchronizing to reference sources of UTC, such as the national time standard maintained by NIST in Boulder, Colorado, known as UTC(NIST). It gets the reliability it needs by automatically switching to backup sources when primary sources fail, allowing the MSCVDC to achieve true fail-safe redundancy.

The MSCVDC has a name that describes its capabilities. Its clock is continuously compared to a reference clock, such as UTC(NIST), using the common-view measurement technique, hence the “CV” part of the acronym. The common-view technique requires the MSCVDC and a receiver connected to the reference clock to each receive, nearly simultaneously, a signal broadcast from the same transmitter. The MSCVDC is a disciplined clock, meaning that its time and frequency are continuously adjusted to be synchronous with the time and frequency of the reference clock, hence the “DC” part of the acronym. Finally, the MSCVDC is a multi-source clock; hence the “MS” part of the acronym. Multi-source capability is both valuable and essential for clocks in critical infrastructure timing systems. Multi-source capability allows the MSCVDC to be disciplined by multiple time scales, with a primary time scale selected by the user and with secondary time scales used as backups to protect against primary time scale failures. It also allows the use of multiple common-view signals, again protecting against failures if a given common-view signal becomes unavailable.

NIST currently distributes UTC(NIST) via its NIST disciplined clock (NISTDC) service,^1^1 Certain commercial equipment, instruments, or materials are identified in this paper to foster understanding. Such identification does not imply recommendation or endorsement by the National Institute of Standards and Technology, nor does it imply that the materials or equipment identified are necessarily the best available for the purpose.
which is a partial implementation of the MSCVDC technique. More than 20 NISTDC units are currently in operation, with several located outside of the United States [4]. A primary application of the NISTDC is the synchronization of stock exchanges and financial markets [5]. The following sections describe the MSCVDC technique in detail, using the partial implementation of the current NISTDC in some examples, but focusing more on what a full implementation of the method can accomplish.

Section 2 provides a technical description of an MSCVDC time distribution system. Section 3 discusses the reliability and redundancy of an MSCVDC distribution system, and Sec. 4 discusses cybersecurity. Section 5 discusses the validation of an MSCVDC as a trusted time reference, and Sec. 6 discusses the accuracy and stability of an MSCVDC with respect to UTC(NIST). Finally, Sec. 7 discusses the feasibility of a commercially available MSCVDC product.

## 2 Technical Description of an MSCVDC Time Distribution System

An MSCVDC time distribution system is based on common-view comparisons of clocks. Before a clock can be synchronized to agree with another clock, the time difference between the clocks must be measured and known. Ideally, the time difference would be measured by direct comparison, after bringing both clocks to the same location. However, if the clocks are geographically separated, for example, if we want to synchronize a clock in Chicago to agree with the UTC(NIST) time scale in Boulder, then direct comparison is not possible. Fortunately, in cases such as this, synchronization can still be accomplished via a common-view comparison.

In our example, a common-view comparison can be arranged if there is a signal that can be simultaneously observed both in Chicago and in Boulder. If such a common-view signal (CVS) exists, then the clocks in Chicago and Boulder can each be simultaneously compared to the CVS. The difference between the two “indirect” comparisons effectively substitutes for a direct comparison and reveals the time difference between the Chicago clock and UTC(NIST). Even though the CVS signal originates from its own clock, the time signal it delivers does not have to be accurate, because it is cancelled out when the two indirect comparisons are subtracted from each other, if the propagation times are equal or if the differences in propagation time can be measured and corrected. In a common-view comparison, the CVS is not the reference clock used for synchronization, but instead just a vehicle that relays time information from one site to another.

**Fig. 1:**
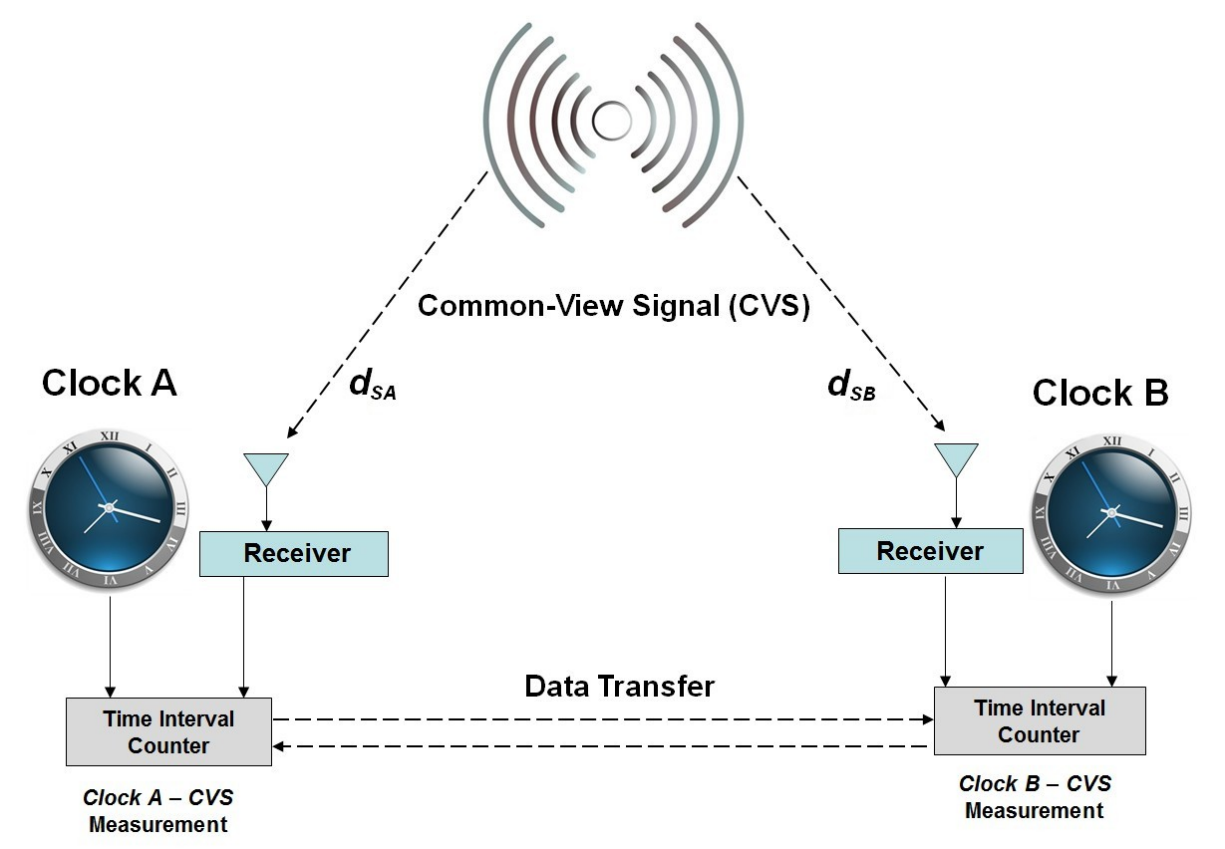
A common-view time transfer system. The system allows accurate time to be transferred from clock A to clock B, without requiring the time signal broadcast by the CVS to be accurate.

Figure 1 shows a common-view time transfer system where a transmitter produces the CVS, and where the CVS is received at sites *A* and *B*. Both sites have a local clock and a receiver that each produce a 1 pulse per second (pps) signal. At each site, the time difference between the received and local 1 pps signals is measured with a time interval counter (TIC). The site *A* measurement compares the CVS received over the path *d_SA_* to Clock A, producing the time difference *Clock A – CVS*. The site *B* measurement compares the CVS received over the path *d_SB_* to Clock B, producing the time difference *Clock B – CVS*.

After the time difference values are obtained, data transfer needs to take place before the measurement results can be processed. Figure 1 shows bidirectional data transfer, but the data transfer could be unidirectional, if only one site needs to know their time difference with respect to the other site. Alternatively, both sites could send their data to a “neutral” site, such as a data repository residing in the cloud, from which each site can retrieve the other site’s measurements. Regardless of how the data transfer is configured, once the two measurements are available, simple subtraction reveals the time difference between the two clocks, because the contribution from the CVS is cancelled out.

To look at this in more detail, delays that are common to both *d_SA_* and *d_SB_* cancel even if they are unknown, but delays that are not common to both paths contribute measurement uncertainty and result in an uncertainty term of *d_SA_* – *d_SB_*. This term represents the relative or differential delay between the two common-view measurement systems. Thus, the basic equation for common-view measurements is

*Clock_A_* – *Clock_B_* = (*Clock_A_* − *CVS*) − (*Clock_B_* – *CVS*) + (*d_SA_* – *d_SB_*). (1)

The delays included in the *d_SA_* – *d_SB_* term can be measured or estimated and applied as a correction to the measurement to reduce the uncertainty. The largest part of both *d_SA_* and *d_SB_* is the propagation delay, or the time interval required for the CVS to travel from the transmitter to the receiver. For radio signals travelling in free space, the propagation delay is nominally equal to the distance divided by the speed of light. If the CVS originates from GPS or from another global navigation satellite system (GNSS), the receiver’s position is determined by the GNSS, and ranging measurements are automatically performed by the receiver to compensate for propagation delay. However, if the CVS originates from a non-GNSS source, such as a geostationary satellite or a terrestrial-based transmitter, and if we can assume that the transmitter position is known, then the position of the receiver must still be independently determined before *d_SA_* and *d_SB_* can be measured and before compensation for the delay differences can be applied.

Once the propagation delay difference has been determined and compensated for, other smaller delays, typically measured in nanoseconds, might still need to be measured or estimated. For example, if the CVS originates from a satellite, delays are added as the signal passes through the ionosphere and troposphere on its way to the Earth’s surface. Other delays are added by multipath signal reflections, and by antenna coordinate errors. After the signal reaches the antenna, delays are introduced by the antenna, antenna cable, and receiver, and these delays must also be measured or estimated (details about how an MSCVDC is calibrated are given in Sec. 5). The goal is to reduce measurement uncertainty as much as possible by making *d_SA_* – *d_SB_* as small as possible, and some common-view systems routinely compare clocks with uncertainties of < 10 ns.

The common-view technique is a passive, receive-only method of transferring time. The CVS travels from the transmitter to the receiver, but the receiver does not exchange messages or interact in any way with the transmitter, thus the transmitter has no knowledge of how many receivers exist, and the potential number of receivers is essentially limitless. As previously noted, data transfer is required before measurement results can be obtained, but because the data stream is not a timing signal, its latency is not important, at least not at the level of microseconds or milliseconds. If the latency can be reduced to seconds or even one minute, common-view becomes a very powerful technique, because that is fast enough to generate corrections and compensate for the frequency drift in a quartz oscillator. Common-view techniques can then be used not only to compare high accuracy atomic clocks, but also to automatically control any clock, in much the same way that GPS signals control a GPS disciplined clock (GPSDC). The next section describes how a common-view disciplined clock (CVDC) works.

### 2.1 Method of Operation for a Common-View Disciplined Clock (CVDC)

**Fig. 2:**
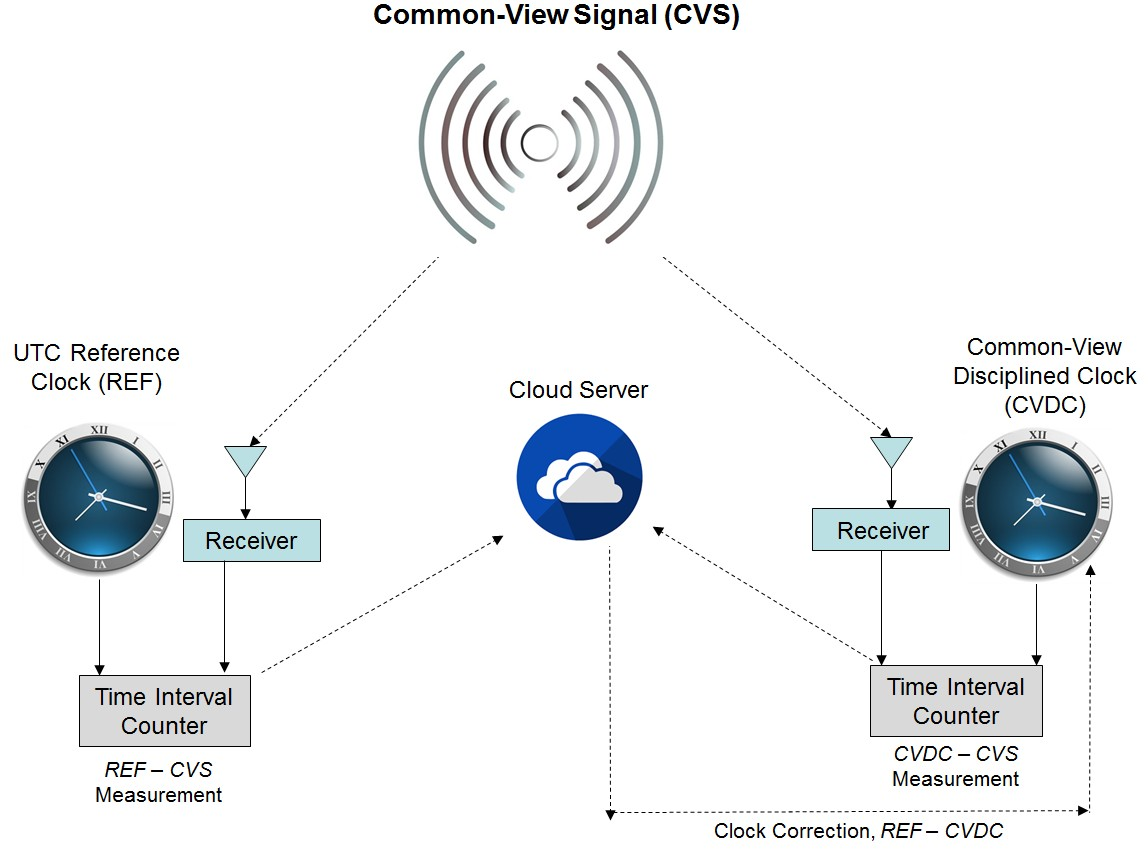
A common-view disciplined clock. Here, the common-view comparisons are used not only to measure a remote clock with respect to a reference clock, but also to control the remote clock. This is done by processing the common-view comparisons in real time, converting them to clock corrections, and then applying the corrections to the remote clock so that it agrees with the reference clock.

A CVDC, an instrument first described in Ref. [6], is a clock for which frequency and time are controlled by a reference source of UTC through the use of common-view comparisons. A block diagram of a CVDC is shown in Fig. 2.

Time from a reference UTC time scale is transferred to the CVDC by use of the common-view disciplining method [3, 5, 6, 7]. As illustrated in Fig. 2, a UTC reference clock, such as the UTC(NIST) time scale in Boulder, Colorado, is compared to a CVS by measuring the time difference *REF* – *CVS*. A simultaneous comparison at a remote site measures the time difference *CVDC* – *CVS*.

Periodically, the UTC reference clock and the CVDC each send their measurement result to a cloud server, where the difference between them, (*REF* – *CVS*) – (*CVDC* – *CVS*), produces the time difference *REF* – *CVDC*. The CVDC then retrieves *REF* – *CVDC* from the server and converts this time difference to a frequency correction by use of an adaptive proportional-integral-derivative (PID) controller or a similar control loop. The frequency correction is then applied to the CVDC’s local oscillator. The process is continuously repeated to keep the CVDC locked to the UTC reference clock.

A major advantage of a CVDC is that any UTC reference clock can control the CVDC if *REF* – *CVS* measurements from the reference clock are made available in real-time. This makes CVDC distribution attractive to national metrology institutes such as NIST that want to distribute their own time scale to remote facilities. NIST has deployed CVDCs as part of its calibration services program since 2010 [7], and similar systems have been subsequently introduced by laboratories in Canada [8, 9], China [10, 11], the European Union [12], Japan [13], Mexico, and perhaps elsewhere.

Despite its versatility and usefulness, a CVDC has vulnerabilities that must be addressed when developing a critical infrastructure timing system. Possible failure modes include a UTC reference clock that is either unavailable or inaccurate, the inability to receive the CVS at either the reference site or the CVDC site, or a network outage at either site. The loss of CVS reception or a network outage for extended periods will cause a CVDC to go into holdover mode and become a free-running clock, just as a GPSDC does when it is unable to receive GPS. Each one of these vulnerabilities is addressed by the MSCVDC.

### 2.2 Method of Operation for a Multi-Source Common-View Disciplined Clock (MSCVDC)

An MSCVDC [3] is an enhanced version of a CVDC that adds multiple layers of redundancy to the basic design. Like a CVDC, it includes a receiver, a time measurement device such as a TIC, a local oscillator, and a network interface. However, unlike a CVDC, its receiver is capable of receiving more than one type of CVS, and its control software is slightly more complex. It delivers on the CVDC’s promise of allowing the user to select between different sources of UTC. It also provides fail-safe layers of redundancy that a CVDC (or a GPSDC) cannot provide. For example:

• If a UTC source is unavailable, the MSCVDC can switch to another UTC source (Sec. 2.2.2).
• If a CVS is unavailable, the MSCVDC can switch to another CVS. For example, it can potentially switch from GPS to another GNSS satellite, to a geostationary satellite, or even to a terrestrial radio or television station. This mitigates concerns about GPS failures (Sec. 2.2.3).
• If a network connection is unavailable, the MSCVDC can directly reference itself to the CVS source without any clock corrections, or perhaps obtain clock corrections from a wireless source (Sec. 2.2.4).

**Fig. 3:**
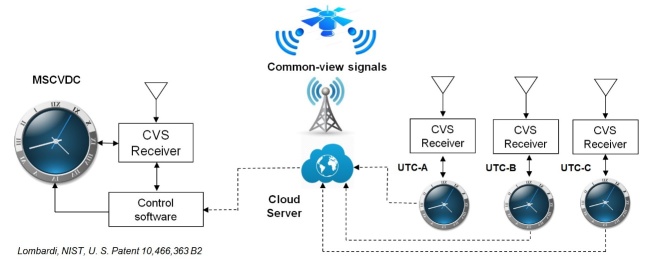
Block diagram of an MSCVDC distribution system. Each MSCVDC has multiple layers of redundancy, including access to multiple CVS sources and multiple reference time scales.

Figure 3 is a simplified diagram of a MSCVDC time distribution system. The right side of the diagram shows that common-view signals are being received at three different locations that maintain UTC, labelled as UTC-A, UTC-B, and UTC-C. In practice, the three UTC sources could be the primary NIST time scale in Boulder, Colorado, and the secondary NIST time scales in Fort Collins, Colorado, and Gaithersburg, Maryland. Other possibilities are discussed in Sec. 2.2.2.

The MSCVDC measures the time difference between the CVS and its local oscillator. At each UTC time scale location, the time difference between the CVS and UTC is measured. The measurements taken at each UTC location are uploaded to a cloud server, shown in the middle of Fig. 3, and then downloaded from the cloud by the MSCVDC. By subtracting the UTC measurements from its own measurements, the MSCVDC now knows its time difference with respect to each UTC source, so it can potentially use any of them for synchronization. The MSCVDC is configured to select one UTC source as its primary source. If the primary source fails or becomes inaccurate, the clock automatically switches to a secondary UTC source. If *N* represents the number of available UTC sources, then *N* – 1 is the number of UTC source failures that a MSCVDC can withstand. A detailed discussion of UTC reference options follows in Sec. 2.2.2.

The middle part of the diagram shows two CVS possibilities, one originating from a satellite and the other originating from a ground-based transmitter. If the CVS is unavailable, the MSCVDC can automatically switch to another CVS. For example, if a multi-constellation GNSS receiver is included, and GPS signals are unavailable, the clock can switch to signals broadcast by another satellite navigation system such as Galileo. If the appropriate receivers are included, the clock can also switch to a CVS that originates from a geostationary satellite, or from a ground-based radio station. If *N* represents the number of available CVS sources, then *N* – 1 is the number of CVS source failures that a MSCVDC can withstand. A detailed discussion of CVS options follows in 2.2.3.

If the network connection fails, the clock can begin using the CVS signal as a reference. For example, if no common-view corrections from a UTC reference are available via the network, the MSCVDC can use any of its CVS sources to discipline its frequency and maintain synchronization until the network connection is restored. If the CVS originates from GNSS, the time steps that are introduced when the MSCVDC switches from common-view comparisons with a UTC reference to direct comparison with a GNSS source will be small, usually much smaller than 0.1 µs, so it is normally not a problem for a ±1 µs requirement. Even so, if the time steps introduced by this type of a switch are a problem, they can be automatically removed, if the MSCVDC kept track of its time difference with respect to both the CVS and the remote UTC reference prior to the network outage.

Because of its ubiquitous nature, the Internet is typically utilized as the network source. However, because so little information (in terms of bytes) needs to be transferred to the MSCVDC, it would be possible to utilize existing direct broadcast wireless sources for the delivery of the common-view corrections. Again, if *N* represents the number of available network sources, then *N* – 1 is the number of network source failures that a MSCVDC can withstand. Section 2.2.4 discusses data transfer options and configurations in more detail.

Table 1 lists the approximate steps that an MSCVDC distribution system performs. Note that the table omits details that are specific to a particular MSCVDC design, such as the averaging interval used to obtain a data point, the frequency of data transfer, the criterion used to establish the validity of a reading, as well as the period of a CVS, reference clock, or network outage that must elapse before the MSCVDC switches to another source. Some details about the considerations taken into account when making these design decisions are provided in Sec. 2.2.2 to Sec. 2.2.4. Before those sections, Sec. 2.2.1 provides some details about the current NIST implementation of an MSCVDC.

#### 2.2.1 Current NIST Implementation of MSCVDC

**Fig. 4:**
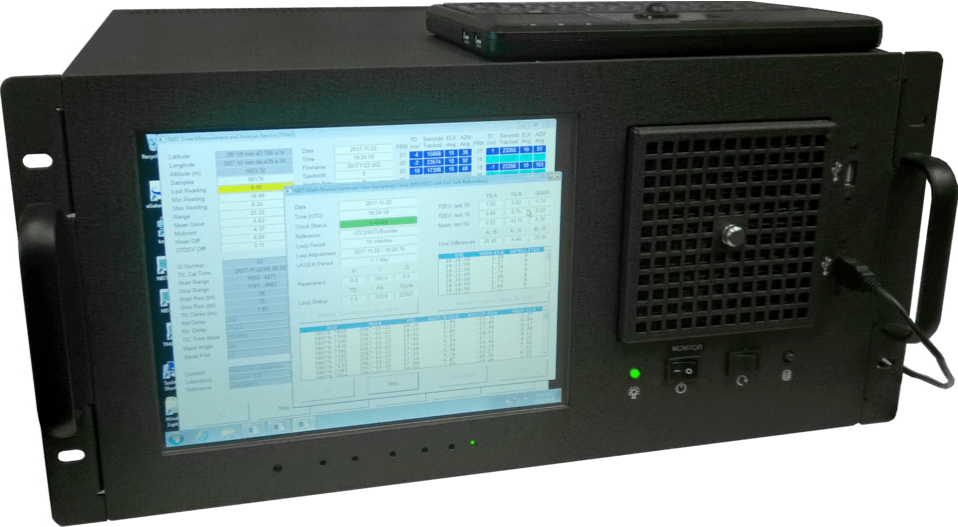
NIST’s implementation of the MSCVDC, the NIST disciplined clock (NISTDC).

NIST currently offers a partial implementation of the MSCVDC to subscribers through the NISTDC service. The service typically utilizes a rubidium clock, but a cesium clock version of the service is also offered [4]. Currently deployed at more than 20 locations, NISTDC units synchronize clocks at several stock exchanges, including the New York Stock Exchange and the Nasdaq. A NISTDC also serves as the reference clock for NIST radio station WWVH in Hawaii. The NISTDC maintains synchronization to within about 10 ns, or 0.01 µs, of UTC(NIST), and is packaged as a rack-mount instrument as pictured in Fig. 4.

**Table 1:** Steps performed by an MSCVDC time distribution system.

**Step**	**Action**
**1**	The MSCVDC compares its local oscillator to *CVS*_1_ to *CVS*_N_ to obtain a series of local time differences, *LTD*_1_ to *LTD*_N_.
**2**	The MSCVDC performs a sequential search of *LTD* series until a valid reading is found.
**3**	The MSCVDC identifies valid *LTD* as MSCVDC – *CVS*_i_, or *LTD*_i_.
**4**	Concurrent with step 1, each UTC site compares its reference clocks to *CVS*_1_ to *CVS*_N_ to obtain a series of reference time differences, *RTD*_1_ to *RTD*_N_.
**5**	Each UTC site, 1 to *N*, uploads their *RTD* series to the network.
**6**	The MSCVDC requests the *RTD* series from the network for the primary and secondary time scales it has selected. If available, skip to step 7. If no network sources are available, convert *LTD*_i_ to a frequency correction and skip to step 9.
**7**	The MSCVDC finds the value in the *RTD* series that corresponds to *REF*_1_ – *CVS*_i_. If valid, this value is identified as *RTD*_i_. If invalid, the MSCVDC performs a sequential search of the RTD series to find a *REF* value that matches the *CVS* used by *LTD*_i_.
**8**	The MSCVDC converts *RTD*_i_ – *LDT*_i_ to a frequency correction.
**9**	The MSCVDC applies the frequency correction to the local oscillator using a PID controller.
**10**	The MSCVDC applies *RTD*_i_ – *LDT*_i_ time correction when necessary to improve “lock” time.
**11**	The MSCVDC stays locked to the UTC reference by repeating steps 1 to 10.

**Fig. 5:**
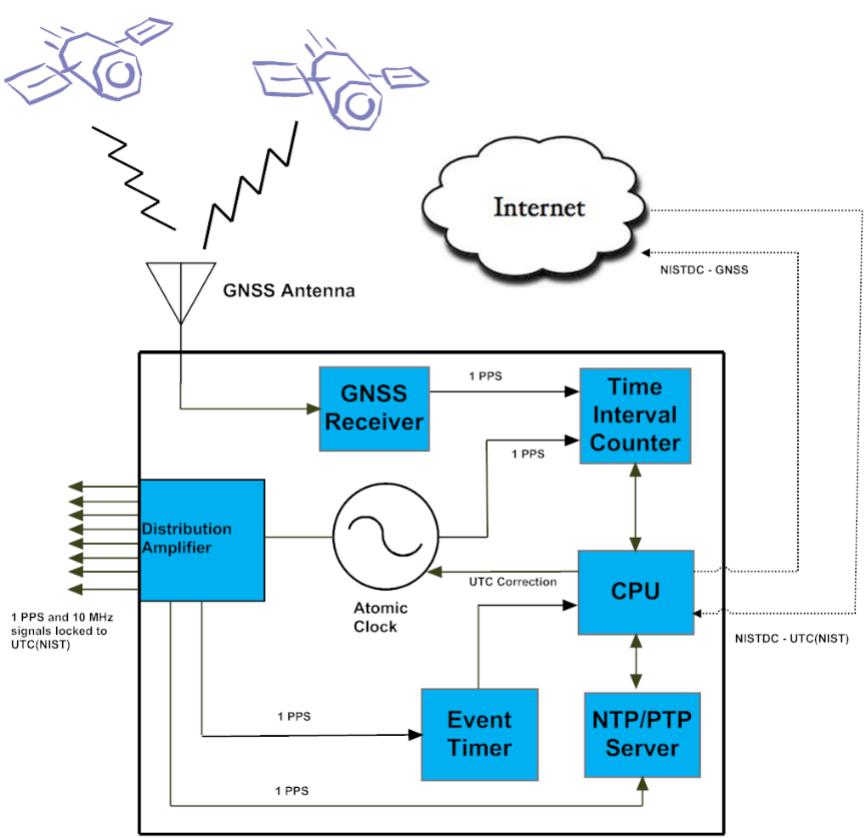
Block diagram of NIST Disciplined Clock (NISTDC).

**Fig. 6:**
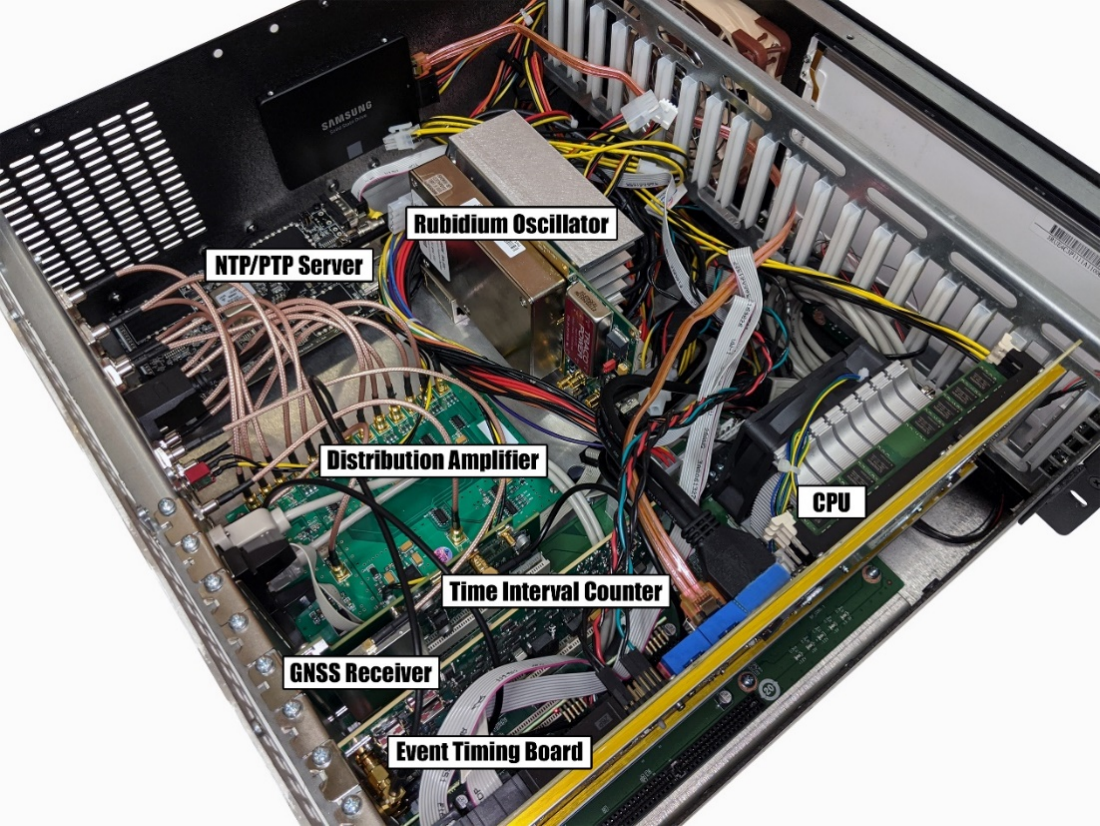
Interior of NISTDC chassis. The visible components include a rubidium oscillator, a time interval counter, a GNSS receiver, an amplifier for distributing clock signals, and other hardware that is utilized to synchronize and measure computer time servers.

The NISTDC houses an internal rubidium oscillator (unless an external cesium clock is used), a GNSS receiver, a TIC, a central processing unit (CPU), a time server synchronized to the NISTDC that supplies time in the network time protocol (NTP) and precision time protocol (PTP) formats, an event timing board that helps monitor the accuracy of local time servers by comparing them to the NISTDC, and a distribution amplifier that produces 10 MHz and 1 pps signals locked to UTC(NIST). Figure 5 provides a block diagram, and Fig. 6 is a photograph of the interior of a NISTDC unit.

#### 2.2.2 Reference UTC Time Scales for MSCVDC

In theory, any UTC reference clock, including all of the UTC(*k*) time scales listed on the International Bureau of Weights and Measures (BIPM) Circular-T [14], could serve as the reference clock for an MSCVDC. The only requirement is that the UTC reference clock must make *REF* – *CVS* data publicly accessible in a format that the MSCVDC can read, using a CVS that the MSCVDC can receive. If these requirements are met, then the MSCVDC can potentially replicate the performance of any UTC reference.

If the goal is to develop a resilient critical infrastructure timing system in the United States, then the reference clock for a MSCVDC should be a U. S. time scale, such as the primary UTC(NIST) time scale in Boulder, Colorado, or the secondary time scales located in Fort Collins, Colorado, or Gaithersburg, Maryland. The other UTC(*k*) time scales located in the United States could also be utilized if permissions were obtained, including UTC(USNO) from the United States Naval Observatory; UTC(NRL), located at the Naval Research Laboratory in Washington, DC; and UTC(APL), located at the Applied Physics Laboratory of John Hopkins University in Laurel, Maryland. Three UTC reference clocks that are geographically separated should be enough to provide fail-safe redundancy, but if sufficient data transfer links are in place (Sec. 2.2.4), there is no technical limit to the number of UTC references that can be included in an MSCVDC design.

**Fig. 7:**
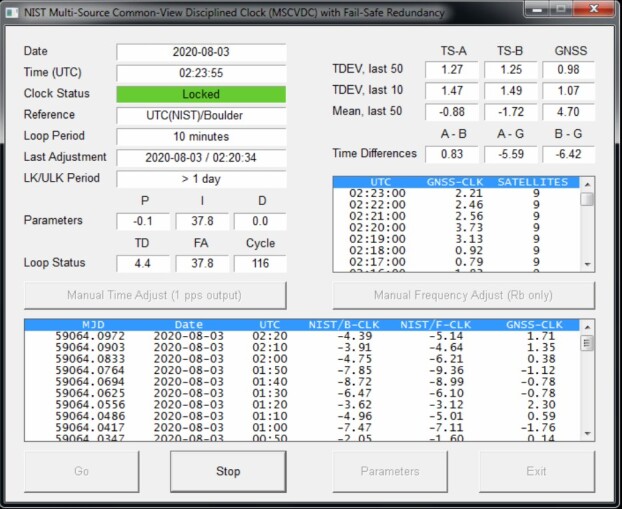
MSCVDC software that controls the NISTDC.

The MSCVDC software used by the NISTDC currently has switchover capability between the primary NIST time scale in Boulder, Colorado, and the secondary time scale in Fort Collins, Colorado. If data from either time scale are unavailable, the GNSS signal used as the CVS becomes the reference source. Figure 7 shows the software in operation at a stock market location.

The large window near the bottom of the display compares the NISTDC, labelled here as CLK, to the primary time scale in Boulder (NIST/B), the secondary time scale in Fort Collins (NIST/F), and the GNSS signal used as the CVS. A new data point is obtained every 10 minutes. The boxes in the upper right corner of the display show the average time differences between the three sources (in nanoseconds) during the past eight hours. In this example, the differences are quite small, where *A* – *B* is the difference between the primary and secondary time scales, or 0.83 ns. The differences between the primary time scale and the GNSS source, or *A* – *G*, and the secondary time scale and GNSS, or *B* – *G*, are both near –6 ns. The time steps introduced when the MSCVDC switches between UTC references will not always be this small, but they are usually much smaller than 0.05 µs (50 ns), and therefore insignificant when maintaining a ±1 µs accuracy requirement. If the time steps introduced by switching reference clocks are considered large enough to be a problem, then they can be removed simply by recording the differences between all of the UTC reference sources during periods when all of them are healthy, and then applying corrections when necessary.

The criteria used for switching from the primary to backup time scale can vary, as they depend upon the hardware used in the MSCVDC design as well as other considerations. When using a rubidium clock, the NISTDC switches to the secondary time scale when the primary time scale has not uploaded data for one hour, as shorter outages will not cause any significant time errors. Neither the primary nor backup time scales will upload data if their time difference from UTC, estimated from cross comparisons at the NIST sites, exceeds 0.05 µs (50 ns). Thus, if reference clock data are available to the MSCVDC, it can be assumed to be accurate to within that specification.

#### 2.2.3 Common-View Signal Sources for MSCVDC

The common-view technique for comparing geographically separated atomic clocks predates GNSS, as signals originating from very low frequency (VLF) and low frequency (LF) time signal stations [15] and from television transmitters [16] were utilized as CVS sources long before satellites. However, the usage of the technique became far more common after the launch of the first GPS satellites in the late 1970s [17], and GNSS signals remain the predominant CVS source, capable of time transfer with uncertainties of < 10 ns.

The use of a multi-constellation GNSS receiver is an efficient way to provide an MSCVDC with multiple CVS signal sources. Numerous manufacturers now offer board-level GNSS timing products that receive BeiDou, Galileo, and GLONASS signals, in addition to GPS, all with a single antenna. The characteristics of these GNSS constellations are summarized in Table 2.

Multi-frequency GNSS timing products, for example, GPS receivers with multi-band capabilities (L1, L2, and L5), are still relatively expensive, as they are manufactured in relatively small numbers and marketed primarily to professional users (for geodetic applications, for example), rather than for use in consumer-grade navigation and timing products. However, multi-constellation timing products with just single-frequency capability for each constellation, for example, those that receive GPS L1, BeiDou B1, Galileo E1, and GLONASS L1 (the frequencies highlighted in blue in the table), are now available for less than $100 in single quantity, making them well suited for integration into a low-cost MSCVDC design. A single antenna, essentially the same size as a GPS-only antenna, usually suffices for multi-constellation receivers (both multi-frequency and single-frequency).

**Table 2:** Signals received by multi-constellation GNSS timing receivers. The frequencies highlighted in blue are available on single-frequency products; all frequencies are available on multi-frequency products.

	**BeiDou**	**Galileo**	**GLONASS**	**GPS**
**Host nation**	China	European Union	Russia	United States
**Altitude (km)**	21 150	23 222	19 130	20 180
**Orbit period (hh:mm)**	12:53	14:07	11:15	11:58
**Orbit inclination angle**	55°	56°	64.8°	55°
**Frequencies (GHz)**	**1.561098 (B1)** 1.589742 (B1-2) 1.20714 (B2) 1.26852 (B3)	**1.57542 (E1)** 1.191795 (E5) 1.27875 (E6)	**~1.602 (L1)^a^** ~1.246 (L2)^a^	**1.57542 (L1)** 1.2276 (L2) 1.17645 (L5)
**Number of satellites**	35	30	24	31

^a^ GLONASS is a frequency division multiple access (FDMA) system, so the satellites use multiple frequencies.

Of course, when GNSS signals are used as a CVS, the same types of GNSS vulnerabilities that can impact GPSDCs can also impact MSCVDCs. For example, the two most common GNSS vulnerabilities, radio frequency (RF) interference and spoofing, can cause a common-view link to either be intermittent or to fail if they occur at either the reference clock or the MSCVDC location.

An interfering RF signal can completely block satellite reception, forcing a GNSS receiver to come unlocked. When this happens, the receiver inside an MSCVDC will either turn off its 1 pps signal, meaning that no common-view data will be available (the preferred practice), or it will begin reporting incorrect clock comparisons. The RF signals that interfere with GNSS time signals can have many spectral characteristics and can originate from many sources, making them difficult to categorize [18]. For example, unintentional RF interference can originate from sources including in-band signals (such as signals reradiated from another GNSS receiver or antenna) and out-of-band signals (radar systems, television stations, microwave data links, and so forth). Intentional interference, known as jamming, occurs when an adversary deliberately blocks reception by broadcasting a signal at or near the GNSS frequency. While jamming constitutes a serious threat, unintentional interference is probably more common. In other words, GNSS signals are often blocked accidentally.

The use of multi-constellation GNSS receivers provides more protection against RF interference than single-constellation receivers, such as a GPS-only device. The more costly multi-constellation, multi-frequency receivers, capable of receiving all of the signals listed in Table 2, actually provide a high level of protection against RF interference, as the frequency diversity of the various GNSS signals makes it unlikely that a single interference or jamming source could block all satellite reception [19].

As noted, multi-constellation, single-frequency receivers (those that receive the frequencies highlighted in blue in Table 2) are better suited for widespread deployment in MSCVDCs because they cost less than multi-frequency receivers. Unfortunately, they offer less frequency diversity; for example, the L1 frequencies for GPS and Galileo are identical, and the BeiDou B1 and GLONASS L1 frequencies are nearby in the spectrum. Even so, they still offer considerably more protection against RF interference than a single-constellation receiver. A recent study demonstrated this by using a GNSS simulator to jam a receiver’s L1-band reception of both GPS and GLONASS. Narrow- and wide-band jamming signals, at various power levels, were alternatively directed at both GPS and GLONASS. By raising power levels high enough, to about −74.8 dBm, it was possible to completely block either GPS or GLONASS reception, but not both. In all scenarios, the combination of GPS/GLONASS allowed at least 40% of the combined number of satellites in view to remain usable [20].

Spoofing is the practice of tricking a GNSS receiver into reporting the wrong time or position. With few exceptions [21], spoofing is not accidental and originates from an adversary who deliberately broadcasts a false signal that the receiver misinterprets as authentic. If it occurred on either end of a common-view link, it would cause a false clock comparison to be reported. Fortunately, researchers have designed many techniques to detect, and defend against, spoofing attacks [22], and recently anti-spoofing algorithms have begun to take more advantage of the redundancy provided by multi-constellation receivers. For example, a receiver tracking the four constellations listed in Table 2 should normally track about 30 satellites at once. This makes it easier to detect false signals in one or more of the constellations, even if the spoofing attack affects an entire constellation [23].

In addition to the methods cited above to combat jamming and spoofing, an MSCVDC that looks at multiple common-view links using different GNSS constellations can potentially do interference and spoofing detection of its own and switch to another CVS whenever necessary.

##### 2.2.3.1 Inclusion of a GNSS CVS in a Resilient Timing Architecture

Because of GNSS vulnerabilities such as jamming and spoofing, the school of thought exists that any use of GNSS, including its use as a CVS, is unacceptable in a resilient time distribution architecture. This viewpoint is supported by the fact that GNSS common-view clock comparisons will stop if all GNSS signals are unavailable.

A second school of thought acknowledges that GNSS is the primary time distribution system for the world, that some usage of GNSS is unavoidable in critical infrastructure timing systems, and that eliminating GNSS entirely will weaken the system and make it less reliable. For example, most of the world’s UTC(*k*) time scales, in the event that they completely lost synchronization, could not be easily synchronized again without relying on some form of GNSS common-view. Therefore, the second school of thought reasons that the use of GNSS as a CVS represents only a partial, rather than a full dependency on GNSS, and that a partial dependency is acceptable. Labelling GNSS common-view as only a partial dependency is based on the fact that a clock being synchronized via common-view is not being synchronized to GNSS time, but rather to the time of the clock located at the other end of the common-view link. In fact, as previously noted, GNSS time does not even have to be correct when used as a CVS, because it is cancelled out in the common-view calculation.

The MSCVDC design is based on a third school of thought, which seems more reasonable than either excluding GNSS from critical infrastructure systems or choosing not to worry at all about a partial dependency. This third school of thought simply reasons that the use of GNSS signals as a CVS is acceptable if a non-GNSS CVS source is also available as a backup or alternative. For example, an MSCVDC can work as a system that relies exclusively on GNSS for common-view signals, either single or multi-constellation, by use of inexpensive receiving equipment and a single antenna. Alternatively, if the appropriate receiving equipment and antenna are included, it could switch to a non-GNSS CVS source whenever necessary. Finally, if complete GNSS independence is a design requirement (to satisfy those who subscribe to the first school of thought), then the MSCVDC could be configured to primarily or exclusively rely on a non-GNSS CVS.

Two non-GNSS CVS sources that could be integrated into an MSCVDC design are geostationary satellites and LF ground-based transmitters, described in the remainder of this section. Both of these CVS options have the advantage of a large coverage area. Other localized line-of-sight signals, such as terrestrial television signals and frequency modulation (FM) radio broadcast stations, could also potentially be utilized, but because their coverage area is limited, they would require a more extensive network of transmitters.

##### 2.2.3.2 Geostationary Satellites

Geostationary satellites are in geosynchronous orbit directly above the Earth’s equator, at an altitude (semi-major axis) of 42 164 km when measured from the center of the Earth. The term geosynchronous simply means that the satellite’s orbital period is the same as Earth’s rotation period. To ensure that a satellite stays above the same point on Earth, a geostationary orbit must be circular, meaning that its eccentricity and inclination angle must both be near zero. Thus, when viewed from a fixed location on Earth, a geostationary satellite is permanently fixed in the same position in the sky, so that an antenna pointed at the satellite can continuously receive the signal. This differs from GNSS satellites, which are only in view for parts of the day, generally during two “flyover” periods. The GNSS satellites circle Earth approximately twice a day at an altitude that is approximately half that of a geostationary satellite, with inclination angles between 55° and 65° (Table 2).

Because common-view time transfer does not require the CVS to be synchronized to a UTC reference, signals from communication satellites, such as the direct broadcast satellites (DBS) used for television, can potentially serve as a CVS. With the method previously shown in Fig. 1, two clocks, *A* and *B*, are each simultaneously compared to a signal from the DBS satellite. The DBS signal can be, for example, an agreed upon pulse in the stream of sync pulses embedded in the video portion of the television signal [24].

**Fig. 8:**
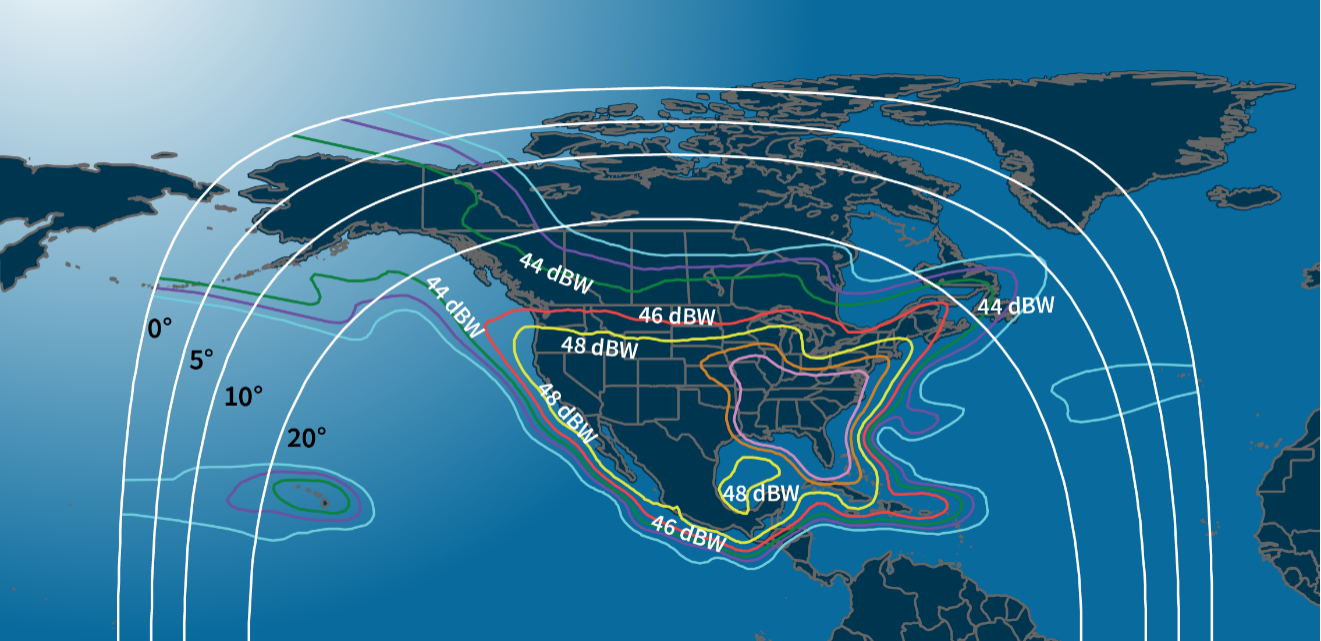
Coverage area of the direct broadcast satellite Echostar 105, located at 105º West longitude, which provides television signals to all 50 U. S. states (courtesy of Echostar).

The coverage area, or footprint, of a geostationary satellite is nearly one third of Earth’s surface. For example, Fig. 8 maps the coverage area of *Echostar 105*, also known as SES-11, a DBS located on the equator at 105º West longitude, essentially on the same longitudinal line as Boulder, Colorado. As the map shows, this position allows *Echostar 105* to provide television coverage to all 50 U. S. states.

When GPS is used as the CVS, the *CLOCK* – *CVS* time differences recorded at each site already include compensation for the propagation delay between the CVS and the receiver. Thus, when the two clock measurements are subtracted, only small, nanosecond-level differences between the two signal paths remain. In contrast, when a DBS is used as the CVS the time differences recorded at both sites will be arbitrarily large, with an uncompensated propagation delay difference between the CVS and each receiver. This typically results in millisecond-level differences between the two signal paths.

To explain why the propagation delays can differ so much between two receiving sites, consider that the height of a geostationary orbit above the Earth’s surface is ~35 786 km above the equator, so the minimum delay occurs if the receiver is located on the equator and the satellite is directly overhead. Applying the speed of light constant (299 792 458 m/s) produces a minimum propagation delay of 119.4 ms. The maximum delay occurs when the receiver is located at the extreme edge of the coverage area, where the satellite appears to be just above the horizon, at an elevation angle near 0º. Then, the distance to the satellite is ~41 756 km, and the propagation delay is 139.3 ms, or about 20 ms more than the minimum delay. Because an MSCVDC could be located anywhere in the coverage area, the propagation delay difference is likely to be multiple milliseconds, with 20 ms representing the worst-case scenario.

Compensating for propagation delay requires knowing the position of both the MSCVDC and the satellite. If the MSCVDC was designed to use GNSS as its primary CVS and to switch to a geostationary satellite when necessary, then its position should already be known, previously obtained from a GNSS position fix. Thus, we can assume that the MSCVDC position is known. The satellite position is more difficult to know accurately because geostationary satellites are not actually stationary but instead trace a repeatable figure-eight pattern in the sky, known as an analemma. The satellite returns to approximately the same position after a period equal to the length of the sidereal day, but during the day the propagation delay of the signal varies within a range of ~200 µs. Because the two stations involved in a common-view comparison simultaneously measure the delay from the CVS, essentially taking snapshots of the satellite from different angles as it traces its figure-eight path, the propagation delay differences for a given measurement will be small compared to the 200 µs variability, and should be at the sub-microsecond level. However, it will still limit the uncertainty of the comparison as a function of the distance between the two stations. Depending on the length of the baseline between the two stations, the propagation delay difference might typically be tens or hundreds of nanoseconds, approaching 1 µs for some baselines, when a constant is used for the satellite position, even if the position of both receivers is known [25]. This type of performance can satisfy a sub-microsecond requirement but will be perhaps an order of magnitude noisier than GNSS common-view time transfer.

Better compensation for propagation delay and hence better time accuracy can only occur if the actual position of the satellite is known in real time and made available to the MSCVDC. This would be possible if the satellite broadcast included updated position data that the MSCVDC could download, which would allow calculation of the propagation delay on the fly, and removal of the propagation delay from the time difference. If no position data are broadcast, the MSCVDC could estimate the satellite’s position itself by using orbital elements downloaded from another source, and then use the calculation results to estimate propagation delay. Satellite providers typically make orbital elements publicly available, but they may only be updated every one or two weeks. The uncertainty of the position calculation will depend upon the age and quality of the orbital elements, with uncertainties increasing as the orbital elements age.

For best results, a precise ephemeris for the geostationary satellite should be generated in real time and made available to the MSCVDC. A method for doing this was proposed by NIST in the 1980s [26] but not implemented. Since then, other similar methods that utilize ground stations and ranging measurements have been demonstrated [24, 25, 27] that can potentially reduce uncertainties to near the GNSS common-view level. Satellite orbit dynamics based on Kalman filters are accurate to about the 1 ns level in direct-range operations to the ground. Regardless of what method is utilized to determine the satellite’s position, once the propagation delay compensation is complete, subtracting the two *CLOCK* – *CVS* values produces the estimated time difference between the two clocks, as given in Eq. (1), which the MSCVDC can convert to a frequency correction and apply to the local oscillator.

Common-view measurement programs via DBS geostationary satellites have been successfully implemented with sub-microsecond accuracy by timing laboratories in India [24, 25], Korea [27], Italy [28], the United Kingdom [29], France [30], China [31], and perhaps elsewhere, demonstrating GNSS independence.

NIST formerly operated a one-way timing service from geostationary weather satellites for many years [32] but has not operated a common-view DBS service. However, the use of a DBS as a CVS holds considerable promise for adding additional redundancy to an MSCVDC, as it makes use of already existing resources. It also provides frequency diversity with respect to GNSS because the downlink frequencies that broadcast television signals to users are typically in the K_u_ band (12 to 18 GHz), with some broadcasts at higher frequencies (for example, in the “B band,” 18.3 to 18.8 GHz, utilized by DIRECTV). An additional directional antenna would be required, but the receiver could likely be made small enough to integrate into the MSCVDC chassis. Table 3 provides just a partial list of DBS satellites that could potentially be used to provide CVS coverage throughout the United States.

**Table 3:** Partial list of direct broadcast satellites that deliver digital television signals to all 50 states and that could potentially provide a CVS source (sorted by longitude).

**Satellite**	**Year Launched**	**Longitude**	**Operator**
**T11 (DIRECTV 11)**	2008	99º W	AT&T
**T14 (DIRECTV 14)**	2014	99º W	AT&T
**T16 (DIRECTV 16)**	2019	101º W	AT&T
**T10 (DIRECTV 10)**	2007	103º W	AT&T
**T12 (DIRECTV 12)**	2009	103º W	AT&T
**T15 (DIRECTV 15)**	2015	103º W	AT&T
**EchoStar 105 (SES-11)**	2017	105º W	EchoStar
**EchoStar X**	2006	110º W	Dish Network
**EchoStar XIV**	2010	119º W	Dish Network
**EchoStar IX**	2003	121º W	EchoStar

##### 2.2.3.3 LF Ground-Based Transmitters

As noted at the beginning of this section, common-view observations using VLF (below 30 kHz), and LF (30 to 300 kHz) radio signals were utilized long before the launch of the first GPS satellite [15]. Later, 100 kHz signals from Loran-C were used in common-view measurements that collected clock data for the UTC calculations. The first use of a GPS common-view link to report to UTC was in 1981 [33], consisting of comparisons between NIST (then called the National Bureau of Standards, or NBS) and USNO. Prior to that, NBS contributed to UTC by directly comparing its time scale to 100 kHz Loran-C signals that enabled a common-view link to the USNO [34].

An experiment conducted in 2003 compared the results of Loran-C common-view to GPS common-view. This was done by using two Loran-C links and a GPS link to simultaneously compare two atomic clocks for a period of two weeks. The clocks were separated by about 445 km, with one located in Gillette, Wyoming, and the other located in Boulder, Colorado. The peak-to-peak variation for the GPS link was ~50 ns, with some variation due to the frequency difference between the two clocks. The Loran-C links were noisier by a factor of about two or three. The first link utilized the Loran-C transmitter in Boise City, Oklahoma, located about 860 km from Gillette and 440 km from Boulder, as the CVS. The peak-to-peak variation was ~100 ns. The second link utilized the Loran-C transmitter in Havre, Montana, as the CVS, a station further away from Boulder than Boise City (~1050 km) but nearer to Gillette (~600 km). In this case, the peak-to-peak variation was ~150 ns. Even though the Loran-C links were noisier, the average time offsets recorded with all three links were within about 10 ns of each other, and the use of Loran-C as a CVS source demonstrated its ability to easily transfer time with sub-microsecond accuracy [35].

In these examples, and in any common-view comparison involving ground-based signals, the paths from the CVS to the two receivers will have unequal propagation delays that will need to be removed before sub-microsecond accuracy is possible. However, as previously noted in the section on geostationary satellites, if the MSCVDC was designed to use GNSS as its primary CVS and to switch to a ground-based station whenever necessary, then its position should already be known, previously obtained from a GNSS position fix. Unlike a geostationary satellite, a ground-based transmitting antenna does not move, so the MSCVDC can simply use a constant value for the CVS coordinates.

If an LF radio signal were employed as a CVS today, it would add tremendous frequency diversity and resiliency to an MSCVDC whose primary links were based on GNSS or geostationary satellite signals. Unfortunately, all of the Loran-C stations in the United States were turned off in 2010. If a revitalized eLoran system returns to the United States, which remains a distinct possibility as of 2021 [36], then eLoran would become a viable ground-based CVS option for an MSCVDC, if an additional receiver and antenna were added to the device.

The only LF signal currently available (as of 2021) for common-view usage in the United States is NIST radio station WWVB (60 kHz). Although WWVB could be utilized as a CVS it has limitations that would make sub-microsecond accuracy difficult, including the cycle ambiguity involved in identifying its on-time marker (OTM), and the fact that it broadcasts from just one location (Fort Collins, Colorado).

#### 2.2.4 Data Transfer and Processing for MSCVDC Distribution System

This section discusses four topics: the current data transfer and processing methods employed by a NISTDC, the data transfer and processing methods recommended for widespread MSCVDC deployment, the frequency of data transfer needed to keep an MSCVDC locked, and methods for providing data transfer redundancy.

##### 2.2.4.1 Data Transfer and Processing Methods Employed by a NISTDC

In the NISTDC implementation of an MSCVDC, data consisting of *NISTDC* – *CVS* measurements are uploaded to an Internet cloud server every 10 minutes using the file transfer protocol (FTP). All NISTDC units have synchronized time-of-day clocks and thus upload data more or less simultaneously, and all files are stored on the cloud server. To prevent unauthorized access, each NISTDC has its own account, with a unique username and password and an Internet protocol (IP) address that NIST has previously safe listed.

Immediately after the upload, the NISTDC issues a request to the cloud server to obtain its common-view time difference. The request is made via the hypertext transfer protocol secure (https). The request is made by issuing a command that follows this basic format:

<NISTDC ID><UTCREF ID><#DATA><CV ID>

where

*NISTDC ID* is a unique code that identifies the NISTDC unit so its data can be retrieved,

*UTCREF ID* is a unique code that identifies the desired UTC reference time scale,

*#DATA* identifies the number of data points requested, and

*CV ID* identifies the CVS source and type of processing (for example, all-in-view or common-view processing can be used with a GNSS CVS source).

The cloud server immediately processes the requested common-view data and sends it back to the NISTDC via https in the form of a time tag followed by a time difference. For example, something similar to this:

**59074.1944#5.49**

where the first five digits are the Modified Julian Date (MJD), followed by the fractional part of the UTC day, a delimiter (#), and the time difference in nanoseconds.

Multiple requests can be issued by the NISTDC to get processed data for more than one server, or more than one CVS, providing the redundancy discussed in Sec. 2.2. As was discussed in that section (Table 1), the received time difference is converted to a frequency correction and applied to the local oscillator to keep the device locked to the desired UTC reference.

##### 2.2.4.2 Data Transfer and Processing Methods Recommended for Widespread Deployment

Note that, in the NISTDC example, the server has copies of all of the data recorded at remote clock sites and that server software performs all of the common-view data processing. This was necessary because NIST has implemented the system as a calibration service to paying customers. As such, NIST must have access to all customer data, to gain knowledge of each NISTDC’s performance. This allows NIST to validate each measurement and to send calibration reports to each customer [4, 5].

A commercial MSCVDC system would handle its data transfer and processing in a different way, similar to a GPSDC. For example, a GPSDC disciplines its local clock with a signal freely provided by the satellites. Providing the signal is the only responsibility of the GPS system. The signal processing is done entirely by the GPSDC. The GPS system has no access to data collected by a given GPSDC and no knowledge of how well a given GPSDC is performing, and of course the GPS system does not issue calibration reports to its users.

The requirement then, for a commercial MSCVDC system, is to have access to freely provided “signals” in the form of *UTCREF* **–** *CVS*_i_ data. After receiving this data, each MSCVDC would perform its own data processing, by making use of its own *MSCVDC* – *CVS*_i_ measurements. This eliminates the step of uploading (pushing) data to a cloud server, but retrieving (pulling) data is still necessary.

Each UTC reference station would provide the necessary “signals” by transferring their common-view data, obtained from multiple CVS sources for resiliency and redundancy, to a data repository, which would likely be an Internet cloud server. From there, the MSCVDC would obtain the data with a request that could be as simple as sending a message code, or for security purposes, sending a message code preceded by an authorization code. The number of possible messages would equal the number of UTC references multiplied by the number of CVS sources. For example, if the primary UTC(NIST) time scale in Boulder and two secondary time scales were each compared to three CVS sources (GNSS, geostationary satellite, and LF radio, for example), the nine possible messages would be:

**<1>UTCREF_1_ – GNSS**
**<2>UTCREF_1_ – GEO**
**<3>UTCREF_1_ – LF**
**<4>UTCREF_2_ – GNSS**
**<5>UTCREF_2_ – GEO**
**<6>UTCREF_2_ – LF**
**<7>UTCREF_3_ – GNSS**
**<8>UTCREF_3_ – GEO**
**<9>UTCREF_3_ – LF**

The number of messages could be expanded; for example, if the three UTC(NIST) time scales provided data from all four major GNSS systems (GPS, Galileo, GLONASS, and BeiDou) instead of just one, the number of messages would expand to 18 (3 × 6). From there, if two additional time scales were added, there would be 30 messages (5 × 6). The MSCVDC could request as many “signals” as necessary to ensure resiliency and to match its own CVS reception capabilities. Requests could be made in a format such as https (compatible with nearly all firewalls), in another existing format, or in a new proprietary format.

##### 2.2.4.3 Frequency of Data Transfer

**Fig. 9:**
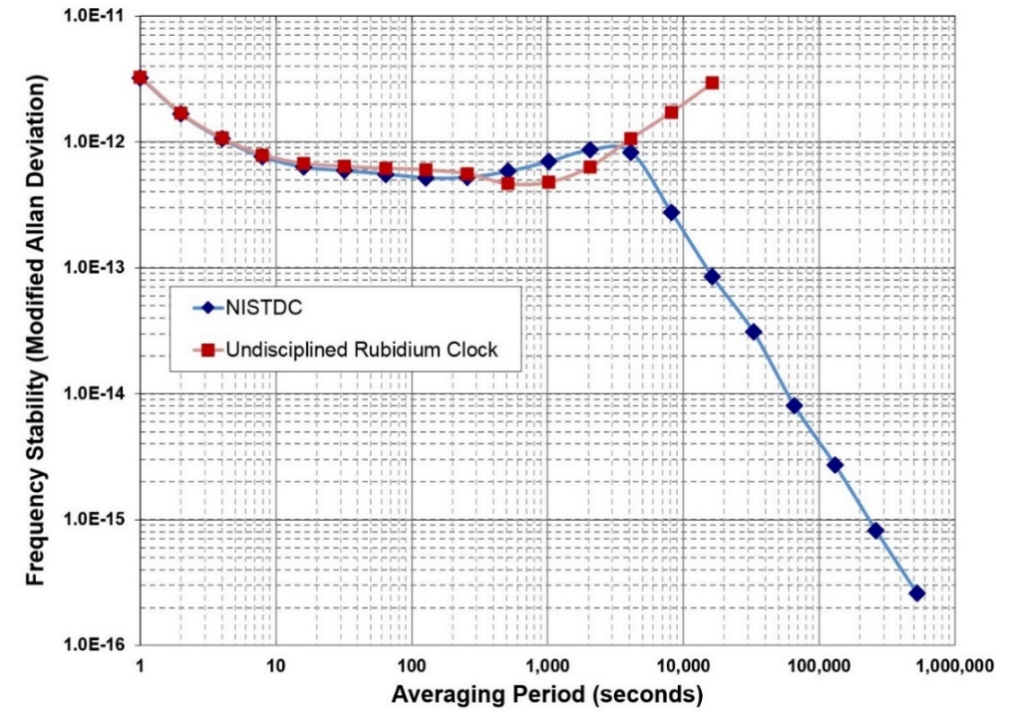
Frequency stability comparison of a free-running rubidium clock to the same rubidium clock disciplined via the MSCVDC method as implemented by a NISTDC. The common-view disciplining improves the rubidium clock performance for all averaging periods longer than one hour.

In the NISTDC implementation of the MSCVDC, new common-view data are made available every 10 minutes. This is sufficient because the NISTDCs have internal rubidium oscillators that are more stable than the common-view time transfer link for this short interval, allowing them to free run without adjustment for more than 10 minutes. To illustrate this, Fig. 9 shows a “crossover” graph, a tool often used to determine how long an oscillator in a disciplined clock can run without requiring a frequency adjustment. The red line shows that the free running rubidium clock reaches a noise floor of near 5 × 10^−13^, as estimated with the Modified Allan deviation at an averaging period of about 1000 s, or ~17 minutes. Due to frequency drift and aging, the clock’s stability with respect to UTC(NIST) then begins to rapidly get worse. The blue line shows the stability of the same rubidium clock when disciplined by a NISTDC. The NISTDC control loop has already taken over before the rubidium noise floor is reached by adjusting the frequency every 10 minutes. This initially makes the stability slightly worse (a longer adjustment interval would be more optimal), but the NISTDC stability drops below rubidium stability after about one hour. For periods longer than one hour, the NISTDC is far more stable, reaching a stability of less than 1 × 10^−14^ after averaging for one day. This stability improves indefinitely, because any frequency differences between UTC(NIST) and a NISTDC are always removed by the common-view corrections.

If MSCVDC units are mass produced, either temperature-controlled or oven-controlled quartz crystal oscillators (TCXOs or OCXOs) are more likely to be utilized than the more expensive rubidium devices, and thus more frequent data transfer will be necessary. This is also the case with low-cost GPSDC designs with inexpensive quartz local oscillators, where the control loop period is usually much shorter than 10 minutes. A GPSDC design, however, has the advantage of a continuously available reference signal, with updates typically available every second, or in some cases even faster.

Although not technically impossible, having a MSCVDC receive updates every second is impractical, and even if a system were designed this way, the common-view data recorded at 1 s intervals would not be especially stable. For this reason, making new common-view data available every minute is recommended. The frequency stability of a common-view link after one-minute of averaging is typically near 1 × 10^−11^.

**Fig. 10:**
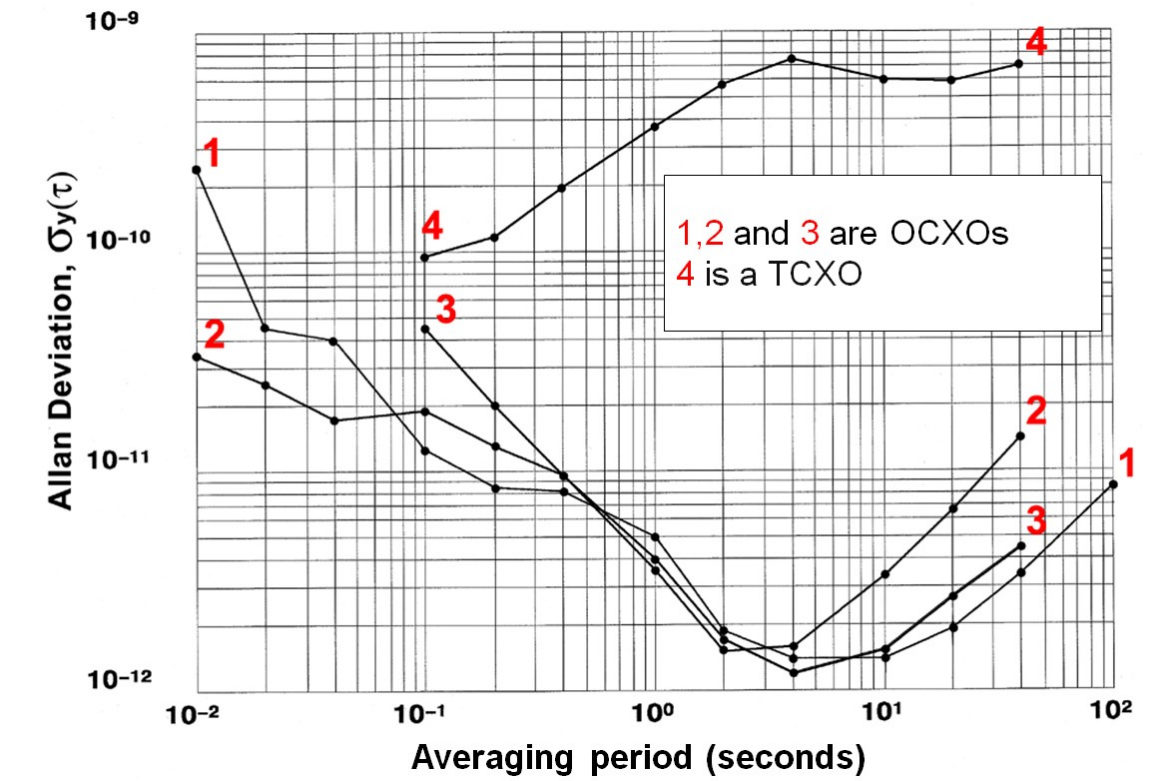
Frequency stability comparison of free-running quartz oscillators.

Figure 10 shows stability measurements performed at NIST for three OCXOs and a poor quality TCXO. This graph does not come close to representing all low-cost local oscillator choices, but it provides some basic guidance. Many low-cost OCXOs, although they typically reach their noise floor in less than 5 s, are still stable to < 1 × 10^−11^ at 60 s, so more frequent adjustments are not necessary. Even a poor quality TCXO should be stable to < 1 × 10^-9^ at 60 s. Therefore, while a 60 s loop period will be too long to maintain the tightest possible TCXO lock, it should limit phase excursions to tens of nanoseconds, and maintain sub-microsecond time accuracy. A 60 s period also provides the flexibility of averaging for multiple minutes and choosing an optimal loop constant for more stable oscillators.

##### 2.2.4.4 Methods for Providing Data Transfer Redundancy

As previously noted, common-view measurements from UTC reference sites are sent to a data repository, which will likely be a cloud server. For redundancy, the same data could be made available from multiple cloud servers, allowing an MSCVDC to switch servers if one is unavailable, or even to cross check data obtained from multiple servers. The servers themselves could be distributed amongst multiple cloud providers (for example AWS, Google Cloud, IBM, Microsoft Azure, and so forth).

Of course, if all local network access is blocked, an MSCVDC will likely need to utilize a CVS as its UTC reference. This could be remedied with a “network-free” MSCVDC if common-view data were made available from a wireless source, which is feasible because only a small number of bytes need to be transferred. Radio signals could modulate the messages described earlier on their carrier frequency, and even if the data transfer rate were slow, the messages could be broadcast via a defined schedule over a period of minutes or hours. Mobile phone signals, signals from radio station WWVB, or signals from a revitalized eLoran system could all potentially provide this service.

## 3 Reliability and Redundancy of a MSCVDC Time Distribution System

The inherent layers of redundancy in a well-designed MSCVDC include multiple UTC reference clocks (Sec. 2.2.2), multiple common-view signals (Sec. 2.2.3), and multiple data links (Sec. 2.2.4), and this combination should provide unmatched reliability. Widespread power outages of long duration represent perhaps the biggest threat to MSCVDC operation, but that is an unavoidable concern with every critical infrastructure system.

**Fig. 11:**
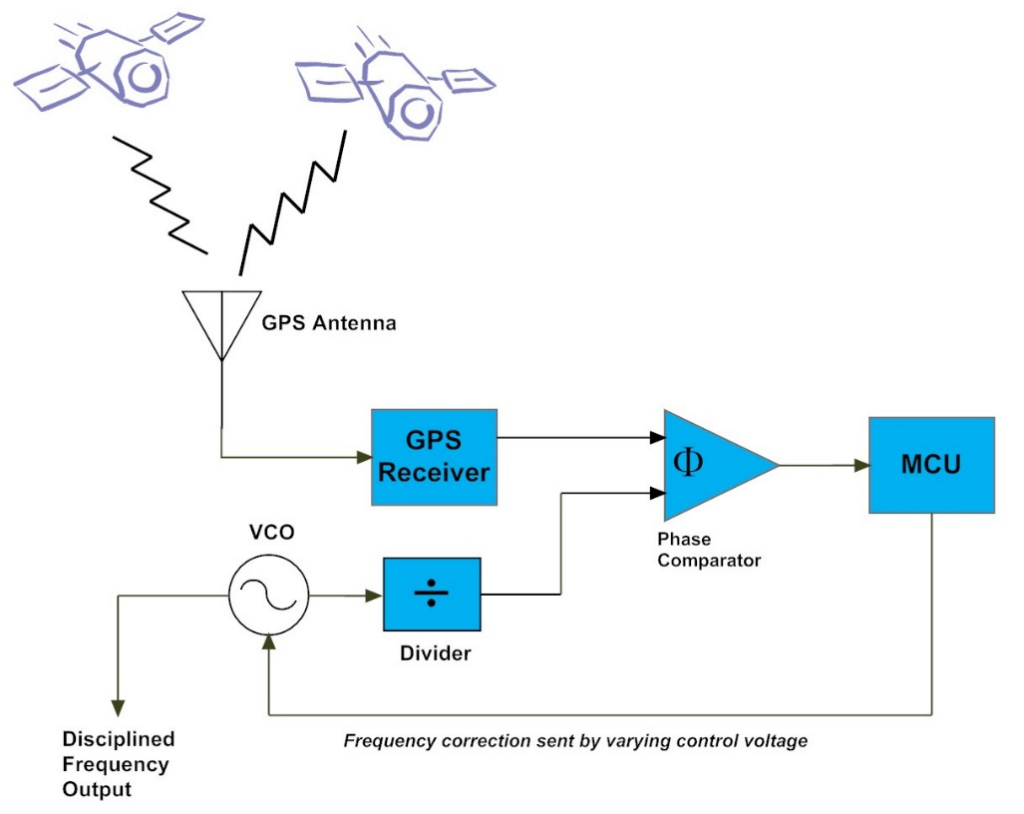
Block diagram of a GPS disciplined clock (GPSDC). The oscillator shown here is a voltage-controlled oscillator (VCO), but various types of local oscillators are included in GPSDC designs.

A reliability question that is certain to be asked involves complexity, because an MSCVDC has more “moving parts” than other disciplined clock systems. For example, a GPSDC, now that the technology has matured and been commoditized, is relatively simple. As shown in Fig. 11, it consists of a GPS receiver and antenna, an oscillator, a phase comparator that compares GPS signals to the oscillator, and a micro controller unit (MCU) that converts the comparison data to frequency and/or phase corrections and sends them to the oscillator, either digitally or as a varying control voltage. This forms a continuously running control loop that locks the oscillator to GPS.

The GPSDC has become ubiquitous, and the electronics and software required to build a rudimentary GPSDC have been refined and commoditized to the point where do-it-yourself (DIY) designs constructed by hobbyists can be found on the Internet. In contrast, a MSCVDC involves systems at multiple locations that share data with each other (Fig. 3), more complex software, as well as cooperation with the institutions that maintain UTC reference clocks.

Even though a MSCVDC is more complex than a GPSDC, it still does not require building any new infrastructure. Instead, it ties together, primarily with software, several mature technologies and resources that already exist. For example, reference clocks like UTC(NIST) are already continuously operating and providing time signals. The freely available CVS signals are already being continuously broadcast for other purposes. The networks and services used to transfer data, such as the Internet and associated cloud services, already exist for a multitude of other purposes.

While the control software of an MSCVDC is more complex than that of a GPSDC, it is still almost trivial when compared to other software systems, such as the e-commerce, news, weather, and messaging applications that we all routinely access on our smartphones. Those systems all have considerably more “moving parts” and are much more difficult to design and understand than an MSCVDC. Even so, they still maintain very high levels of reliability with only infrequent outages. Just as those systems offered a more modern approach to shopping, communications, and information retrieval systems, with benefits that far outweigh their complexity, the MSCVDC potentially offers a more modern approach to resilient time distribution systems than has previously existed.

There would, of course, still be a “learning curve” associated with the development and testing of a MSCVDC software platform. However, it seems likely that such a software platform could rapidly mature, and that any reliability concerns based on complexity could rapidly go away.

## 4 Cyber Security of a MSCVDC Time Distribution System

Raw or processed measurement data resulting from time measurements are not considered to be confidential or proprietary information. This type of data, while not always published in real time, is routinely published by NIST and many other laboratories. While a secure, encrypted data channel, such as https, should be used for data transfer to avoid tampering, it is probably not necessary to require an MSCVDC requesting common-view data to provide an access or authorization code. The number of bytes transferred per request is small, comparable to the data transferred when accessing a public Network Time Protocol (NTP) server, and a standard protocol could be developed for common-view data that allowed a variety of MSCVDC designs to freely access the data and obtain synchronization to UTC in much the same fashion that a GPSDC freely accesses GPS signals.

If cloud servers are utilized as common-view data repositories, two potential concerns are denial of service attacks caused by overloading a server with false requests from non-MSCVDC sources, or hacking into a server and modifying or corrupting the common-view data. Standard security practices routinely used by web servers, NTP servers, and other data providers can guard against these events, as can having an MSCVDC cross checking data obtained from multiple servers.

## 5 Validation of an MSCVDC as a Trusted Time Reference

In the NISTDC implementation of an MSCVDC (Sec. 2.2.1), every instrument, with its associated antenna and antenna cable, is calibrated at NIST in Boulder, Colorado, before it is shipped to the customer. The purpose of the calibration is to measure the differential delay between the customer’s MSCVDC, known as the system under test, and the reference system. The calibration is performed via the common-clock method, where the system under test and the reference system are both measuring the same clock, in this case a 1 pps signal from UTC(NIST). The cables that connect both the reference system and the system under test to UTC(NIST) are carefully calibrated with an uncertainty of about 0.1 ns, so that their differential delay is as close to zero as possible, and the antennas for both systems are mounted on poles that have coordinates known to within 20 cm.

The calibrations last for about one week. When completed, the average time difference of the MSCVDC with respect to the reference system, *D*_Rx_, is entered into the MSCVDC software to compensate for its internal delays [5]. If multiple CVS sources are used, common-clock calibrations should be performed for each CVS, with a separate value of *D*_Rx_ used for each common-view link.

**Fig. 12:**
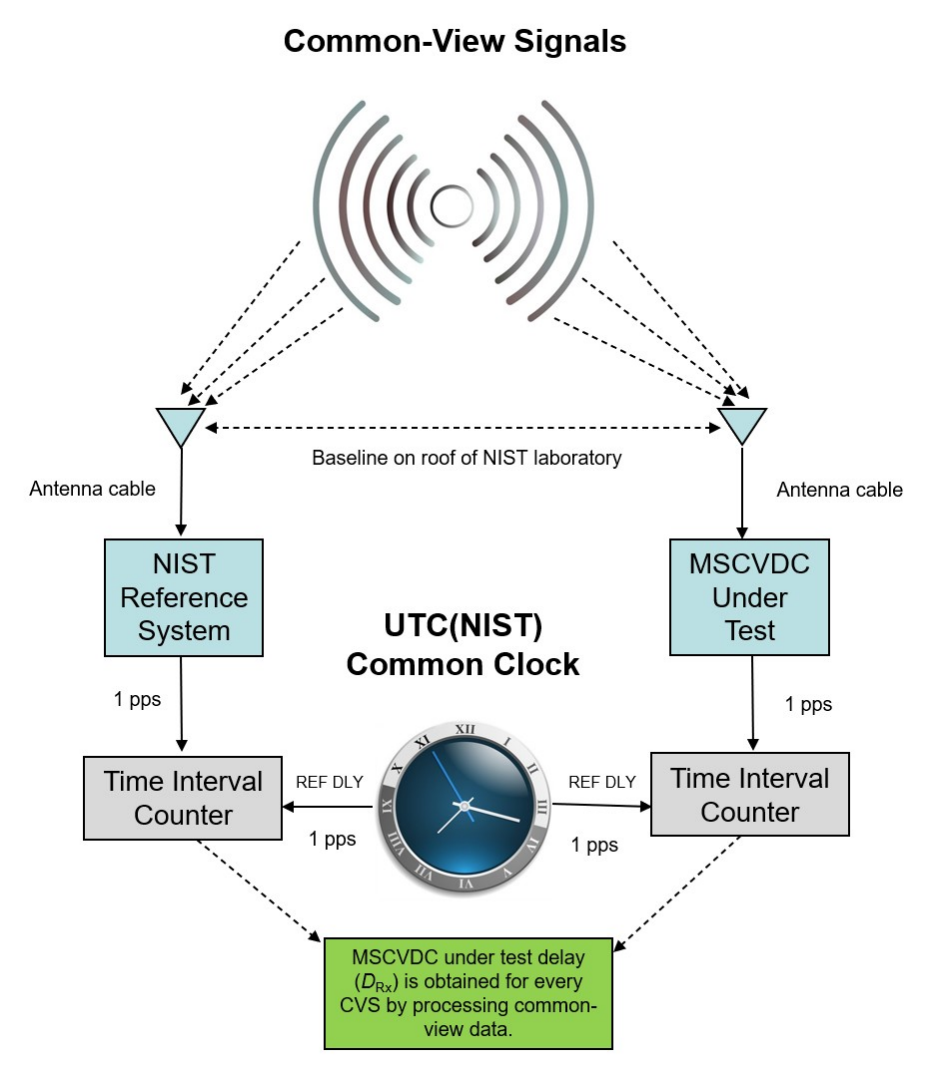
Configuration for a common-clock MSCVDC calibration at NIST.

When the common-clock calibration (Fig. 12) is finished and a delay value has been entered, the 1 pps connection from UTC(NIST) is removed, and the newly constructed NISTDC locks to UTC(NIST) via common-view comparisons. Then, a 1 pps output from the NISTDC is directly compared to the 1 pps output of UTC(NIST) with a TIC, allowing the results of the common-view comparison to be compared to the results of the direct comparison. This is done to ensure that the output of the new NISTDC is within 10 ns of UTC(NIST), which is the uncertainty of the calibration service. Typically, the difference is < 5 ns, and if the unit is outside of its stated uncertainty, it is not delivered to a customer until the uncertainty is brought within specifications.

**Fig. 13:**
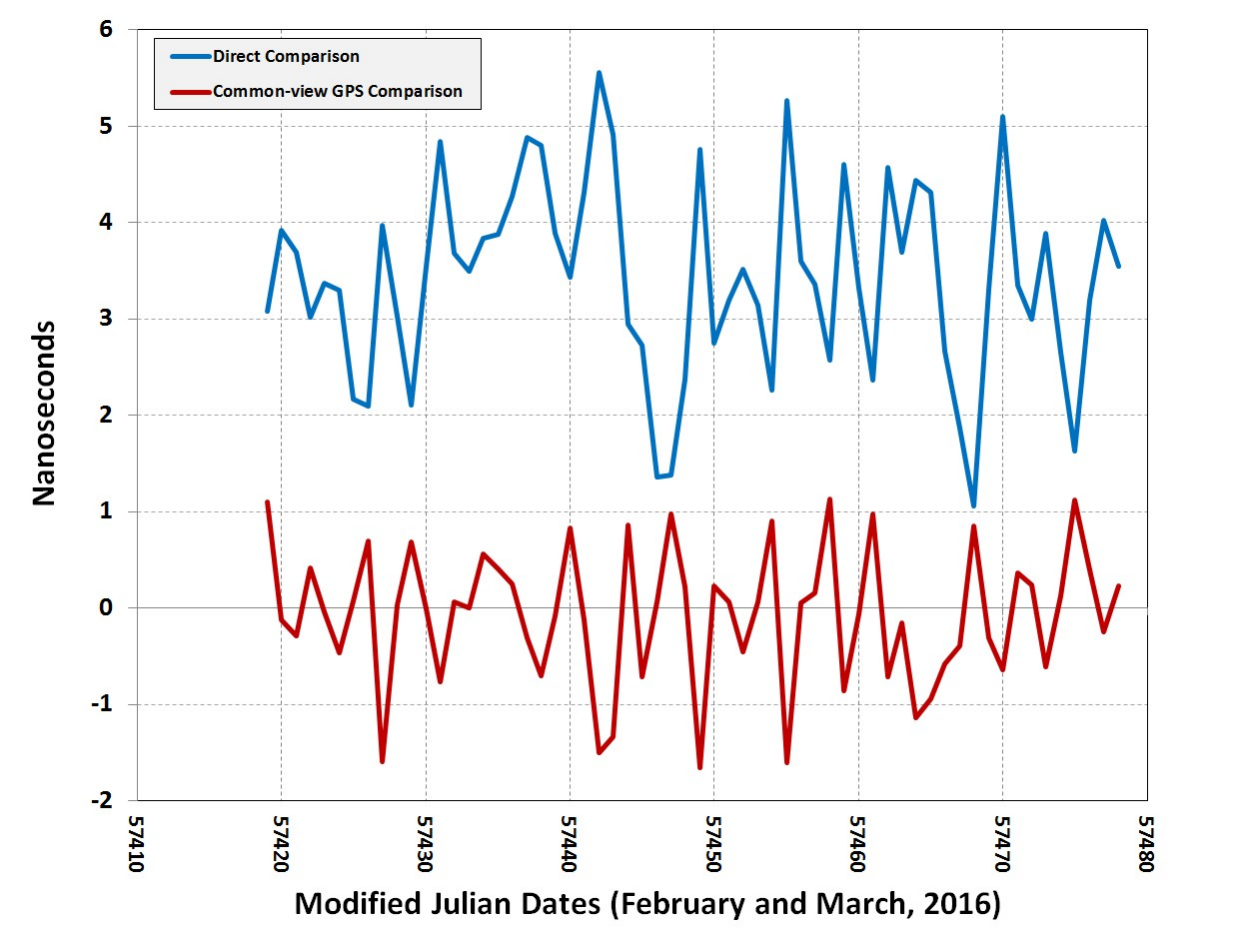
Direct and common-view comparisons of a NISTDC.

To illustrate this, Fig. 13 shows the results of a 60-day comparison between a NISTDC and UTC(NIST). The blue line shows the results of the direct comparison, and the red line shows the results of the common-view comparison. For the 60-day interval, the average value of *NISTDC – UTC(NIST)* was −0.1 ns for the common-view comparison, and 3.4 ns for the direct comparison, a difference of 3.5 ns. This difference was primarily due to biases introduced by the uncertainty of the common-clock calibration, but other factors, including environmental changes since the time of the calibration, might have also contributed to the difference. The common-view comparison will always produce a time difference near 0 because the NISTDC control loop continuously adjusts its clock towards a set point of 0, providing compensation that can hide biases in the calibration. The direct comparison reveals these biases by measuring the true time difference and validates that the NISTDC is operating within its stated uncertainty [5].

This process provides the ultimate validation of an MSCVDC as a trusted time reference, because it actually verifies that the claimed measurement uncertainty is true. It is analogous to bringing a calibrated GPSDC inside the USNO laboratories and comparing it directly to UTC(USNO). However, for widespread deployment of an MSCVDC, it would be unrealistic to individually calibrate every unit, just as it is unrealistic to calibrate every GPSDC.

When GPSDCs are deployed, the premise is that sub-microsecond accuracy is expected without calibration, and this same premise could be applied to a MSCVDC to provide a realistic validation solution. This could be done, for example, by developing a standard configuration for a MSCVDC, one where each unit is constructed with identical hardware and software, and then calibrating a few of the units. All units could then be shipped with pre-inserted *D*_Rx_ values for each CVS source, based on delays for standard antenna cable lengths. As is the case with GPSDC products, the MSCVDC software would allow the *D*_Rx_ values to be changed when necessary, allowing users to enter new values if, for example, longer antenna cables were added.

The uncertainty assigned to the device could be expanded to conservatively cover all situations, again following the GPSDC model. For example, a 100 ns uncertainty (a typical GPSDC specification) should conservatively cover all of the GNSS CVS sources, even when the delay differences between units differ by tens of nanoseconds. Larger uncertainties may need to be assigned to non-GNSS CVS sources, but enough margin remains between 0.1 µs (100 ns) and 1 µs to validate sub-microsecond accuracy requirements for all configurations.

## 6 Accuracy and Stability of an MSCVDC with Respect to UTC(NIST)

**Fig. 14:**
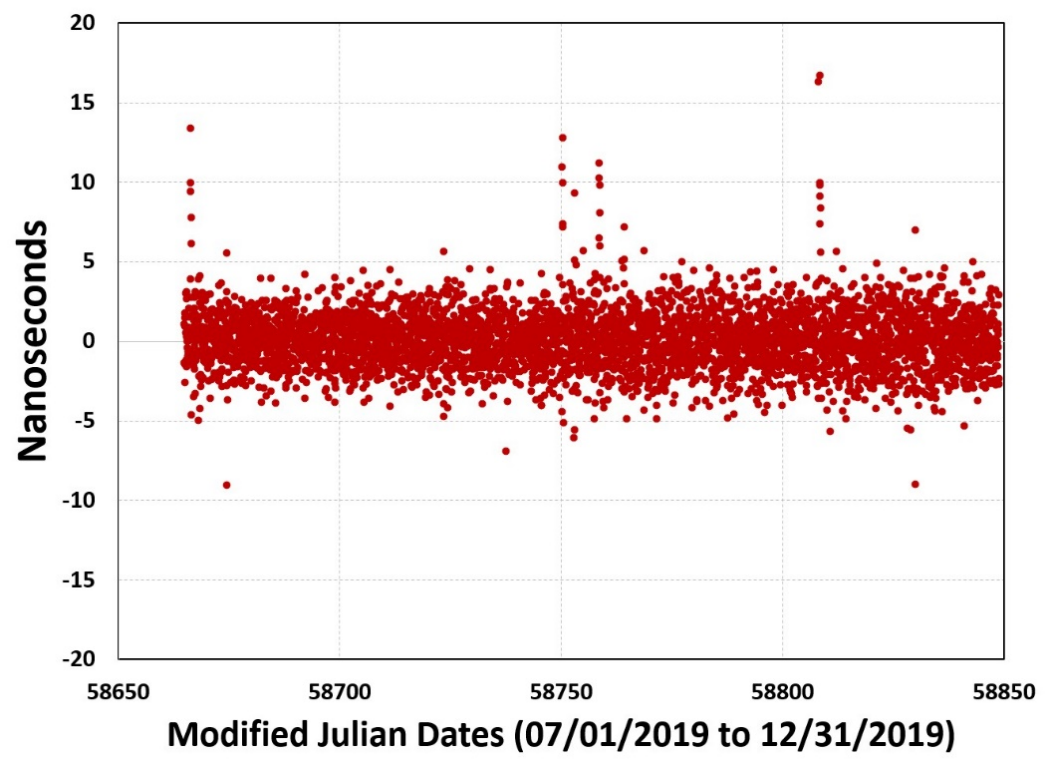
Accuracy of MSCVDC at a major U. S. stock exchange, with respect to UTC(NIST).

A locked MSCVDC, referenced to UTC(NIST) and using GNSS as the CVS, will stay accurate to within a few nanoseconds of UTC(NIST) and report an average time offset near 0. To illustrate this, Fig. 14 shows a 6-month (July to December 2019) comparison of an MSCVDC located at a U. S. stock exchange to UTC(NIST). The data points shown in the figure are one-hour averages. The peak-to-peak variation over the 6-month interval is ~25 ns, but most data points fall within a ±5 ns range, and the average time offset is less than 0.1 ns, or essentially 0 [37].

As was discussed in Sec. 5, the time difference will not actually be 0, but instead will have a small amount of bias introduced by the uncertainty of the MSCVDC calibration and other factors, such as temperature changes that can impact the local oscillator. These small biases are unknown to the MSCVDC control loop, which simply uses the common-view data to continuously adjust the clock towards a set point of 0. For these reasons, the time differences reported by the MSCVDC have an uncertainty of about 10 ns (±0.01 µs) [5].

The accuracy of an MSCVDC will of course be affected by time steps that occur during time scale or CVS switchovers, none of which occurred in the data set shown in Fig. 14. However, if these switchovers had occurred in that data set, they may not have been obvious, as their magnitude is typically a small number of nanoseconds. For example, when a MSCVDC switches to or from a primary to a secondary UTC reference, it will incur a time step equal to the difference between the two references. Thus, if the current time difference between the primary UTC(NIST) time scale in Boulder and the secondary time scale in Fort Collins is 10 ns, and the MSCVDC is forced to switch to the Fort Collins scale because data from Boulder are unavailable, the MSCVDC will incur a 10 ns time step. Switching between CVS sources will also introduce time steps, but if each CVS channel has been calibrated as described in Sec. 5, those time steps should be small, at the level of a few nanoseconds.

The interval selected before an MSCVDC switches from one time scale to another or from one CVS source to another will depend on the stability of the oscillator. For example, if a temporary network outage occurs, and the MSCVDC cannot receive corrections from Boulder for 30 minutes, it may not be necessary to switch to Fort Collins if the local oscillator is a rubidium oscillator. This is the case with the current NISTDC implementation of the MSCVDC, where no switchovers occur in less than 30 minutes. However, if the local oscillator is a TCXO, the switchover will need to occur more quickly.

**Fig. 15:**
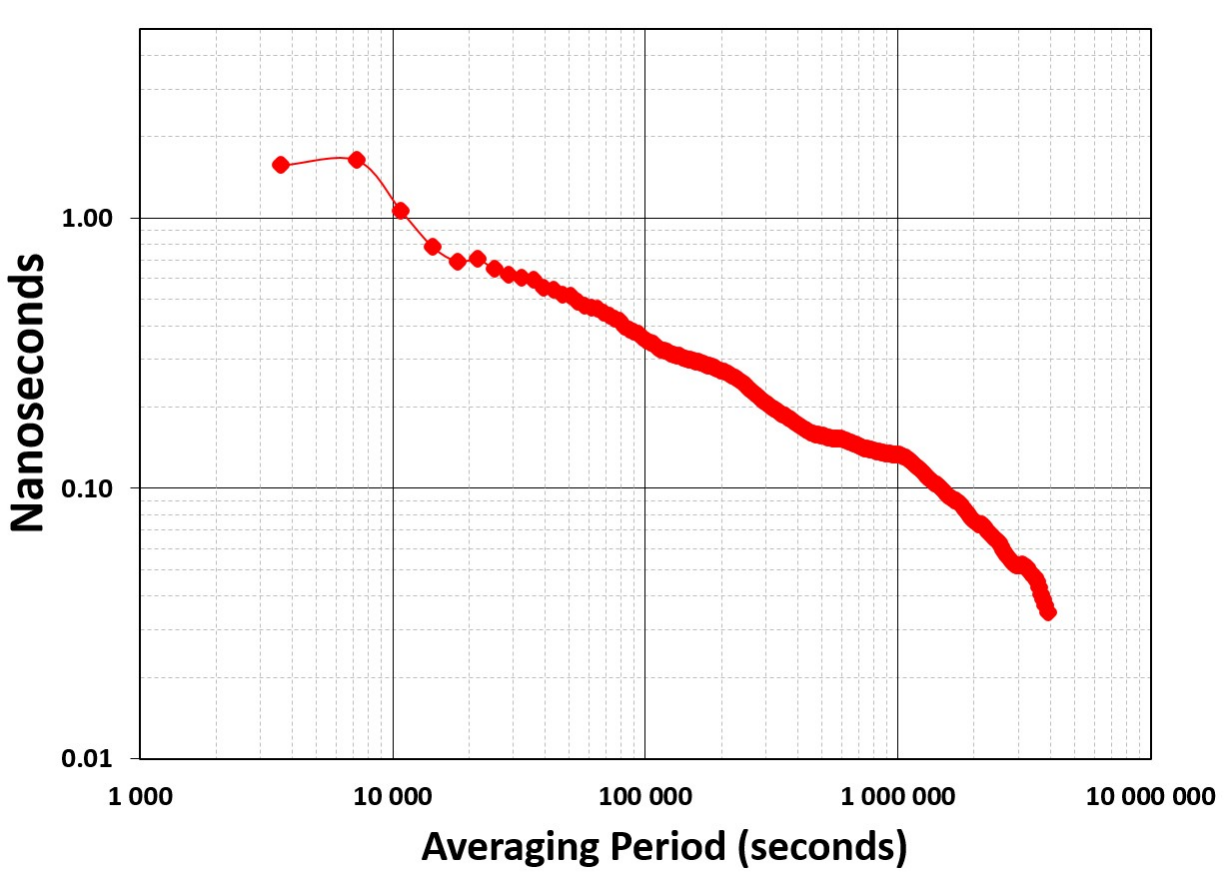
Stability of MSCVDC at a major U. S. stock exchange with respect to UTC(NIST).

Figure 15 shows the time deviation (stability) of the data shown in Fig. 14 for averaging periods ranging from one hour to about one month. After averaging for one hour, the stability is about 1.5 ns, dropping below 0.4 ns after one day and below 0.2 ns after one week. This high level of stability is possible because the time differences between UTC(NIST) and the MSCVDC were always compensated for by the common-view corrections [37].

## 7 Feasibility of a Commercially Available MSCVDC Product

The development and introduction of a commercially available MSCVDC product and/or service are entirely feasible, based on the following factors. The hardware would be similar to existing GPSDC designs with multiple receivers/antennas added for the reception of multiple CVS sources, and network connectivity added for the retrieval of common-view data from UTC reference stations. If the device were to be designed to only have multi-constellation GNSS ability, just one antenna would be necessary. The cost of manufacturing the unit would likely be most heavily influenced by the quality and cost of the internal oscillator, as commoditization has already occurred with most of the other components, and their prices have already benefited from the economy of scale. Perhaps most importantly, the development of commercially available MSCVDC devices is feasible because it would not require new time scales, broadcast stations, or networks to be developed because the necessary infrastructure is already in place.

The most significant investment would likely involve software development. The device software, as previously discussed, would be more complex than GPSDC software but not prohibitively so, and certainly much less complex than the software found in many other commercial instruments and application platforms.

The manufacturer of a commercial MSCVDC product would of course need to collaborate with NIST and/or other government laboratories that maintain UTC time scales, so that reference stations could be installed at those laboratories. The UTC providers would likely be willing to accommodate these reference stations, realizing that the MSCVDC is a convenient and easy way to distribute their time scales to a potentially large number of users. The arrangements with the UTC reference laboratories would likely involve a signed agreement, such as a cooperative research and development agreement (CRADA) or a memorandum of understanding (MOU). The installed reference stations are essentially just another MSCVDC unit, modified to make their data available through a network.

The MSCVDC manufacturer would also need to provide one or more data repositories for common-view data collection, which would likely be hosted by an Internet cloud server provider. Once this basic network infrastructure is provided, a MSCVDC time distribution system could support a nearly limitless number of clocks, with each clock having verifiable accuracy as well as fail-safe redundancy.

## 8 Conclusions

The MSCVDC is a device that can support and strengthen critical infrastructure timing systems by providing them with an accurate (sub-microsecond), reliable, verifiable, and redundant source of time. The layers of redundancy in a well-designed MSCVDC would include multiple UTC reference sources, multiple common-view comparison links, and multiple network links, providing reliability unmatched by any GPS clock. The multiple UTC reference sources allow legal and regulatory time requirements to be met for any application. For example, if an application requires NIST time, or time from another organization to be distributed, the MSCVDC can be configured to meet the requirement.

The development of commercially available MSCVDC devices is feasible and would not require new time scales, broadcast stations, or networks to be developed because the necessary infrastructure is already in place. The most significant investment would likely involve software development, and implementation would also require establishing collaborative agreements with laboratories that maintain UTC time scales.
